# Discovery of new therapeutic targets in ovarian cancer through identifying significantly non-mutated genes

**DOI:** 10.1186/s12967-022-03440-5

**Published:** 2022-05-26

**Authors:** Halema Al-Farsi, Iman Al-Azwani, Joel A. Malek, Lotfi Chouchane, Arash Rafii, Najeeb M. Halabi

**Affiliations:** 1 College of Medicine, Qatar University, Doha, Qatar; 2 Integrated Genomics Core, Sidra medicine, Doha, Qatar; 3 Genomics Core, Weill Cornell Medicine in Qatar, Education City, Qatar Foundation, Doha, Qatar; 4 Genetic Intelligence Laboratory, Weill Cornell Medicine in Qatar, Education City, Qatar Foundation, Doha, Qatar

**Keywords:** RNAi, Epithelial ovarian cancer, RNA-Seq, Non-mutated genes, Unmutated genes, Cancer somatic mutation, Mutated genes, Simulated mutation

## Abstract

**Background:**

Mutated and non-mutated genes interact to drive cancer growth and metastasis. While research has focused on understanding the impact of mutated genes on cancer biology, understanding non-mutated genes that are essential to tumor development could lead to new therapeutic strategies. The recent advent of high-throughput whole genome sequencing being applied to many different samples has made it possible to calculate if genes are significantly non-mutated in a specific cancer patient cohort.

**Methods:**

We carried out random mutagenesis simulations of the human genome approximating the regions sequenced in the publicly available Cancer Growth Atlas Project for ovarian cancer (TCGA-OV). Simulated mutations were compared to the observed mutations in the TCGA-OV cohort and genes with the largest deviations from simulation were identified. Pathway analysis was performed on the non-mutated genes to better understand their biological function. We then compared gene expression, methylation and copy number distributions of non-mutated and mutated genes in cell lines and patient data from the TCGA-OV project. To directly test if non-mutated genes can affect cell proliferation, we carried out proof-of-concept RNAi silencing experiments of a panel of nine selected non-mutated genes in three ovarian cancer cell lines and one primary ovarian epithelial cell line.

**Results:**

We identified a set of genes that were mutated less than expected (non-mutated genes) and mutated more than expected (mutated genes). Pathway analysis revealed that non-mutated genes interact in cancer associated pathways. We found that non-mutated genes are expressed significantly more than mutated genes while also having lower methylation and higher copy number states indicating that they could be functionally important. RNAi silencing of the panel of non-mutated genes resulted in a greater significant reduction of cell viability in the cancer cell lines than in the non-cancer cell line. Finally, as a test case, silencing ANKLE2, a significantly non-mutated gene, affected the morphology, reduced migration, and increased the chemotherapeutic response of SKOV3 cells.

**Conclusion:**

We show that we can identify significantly non-mutated genes in a large ovarian cancer cohort that are well-expressed in patient and cell line data and whose RNAi-induced silencing reduces viability in three ovarian cancer cell lines. Targeting non-mutated genes that are important for tumor growth and metastasis is a promising approach to expand cancer therapeutic options.

**Supplementary Information:**

The online version contains supplementary material available at 10.1186/s12967-022-03440-5.

## Background

High-grade serous ovarian carcinoma (HGS-OvC) is the most lethal gynecological cancer with around 22,000 new cases and 14,270 deaths per year in the United States. It is ranked 5th overall for cancer death in women [1–3]. The Cancer Genome Atlas (TCGA) program performed a comprehensive “omics” characterization of HGS-OvC (TCGA-OV). They studied 489 ovarian cancer samples integrating copy number variations, transcriptomic, methylation arrays, and micro-RNA expression data and performed exome sequencing for 316 of the samples [4]. Patients in the TCGA-OV project had advanced primary ovarian cancer with 5% diagnosed at stage 2, 79% at stage 3 and 16% at stage 4.

TCGA-OV researchers identified mutations that are important in ovarian tumors by comparing pathogenic variants to those found in the Catalogue of Somatic Mutations in Cancer (COSMIC) and Online Mendelian Inheritance in Man (OMIM), and by predicting the mutations’ impacts on protein function. The TCGA-OV study further analyzed the significance of all mutated genes compared to the background mutation rate (BMR), which represents the rate of random mutation. These estimates assume that most observed mutations are neutral and don’t have any selective advantage or disadvantage [5, 6].

Estimating the significance of gene mutation done by the TCGA-OV research network relied mainly on frequency-based criteria, where a gene is identified as having a driver mutation if it is altered in significantly more patients than expected based on the background model. Mutations in some genes, such as *TP53*, are detected in large populations of different cancers whereas some mutations exhibit low rates in cancers. For each gene, they calculated the probability of seeing the observed set of mutations and reported nine significant mutations out of the 9986 observed mutated genes. *TP53* was found to be mutated in more than 96% of all samples as previously reported [7–10]. BRCA1/2 variants were also found in 22% of tumors (a combination of germline variation and somatic mutations). The TCGA-OV group also identified significantly mutated genes that occur at a low frequency, in only 2–6% of tumor samples. These genes are *RB1*, *NF1*, *FAT3*, *CSMD3*, *GABRA6*, and *CDK12* [4].

While characterizing the spectrum of somatic mutations in ovarian cancer in the TCGA-OV study has a high impact, cancer arises from a complex interplay between genes in cells and environmental factors [11] and both mutated and non-mutated genes interact to enable the acquisition of the hallmarks of cancer [12]. Understanding which non-mutated genes are important for tumors could lead to the development of new and more effective drug targets. Most studies have focused only on mutated genes because it is difficult to assign significance to non-mutated genes since most genes in a single patient would be non-mutated. However, using high-throughput sequencing data of many patients, it is possible to estimate the significance of non-mutated genes by comparing observed mutation frequencies to expected mutation frequencies and identifying genes with lower mutation frequencies than expected.

In this study, we used a computational biology approach and set up in-silico mutagenesis experiments. This allowed us to identify a subset of genes that were observed to have fewer mutations in observed data than expected from simulation data which we called non-mutated genes. We hypothesized that non-mutated genes were essential to tumor function. Pathway analysis showed that non-mutated genes interact in cancer-related pathways. Gene expression studies showed that non-mutated genes were well-expressed in cell lines and ovarian cancer tissues from patients. We also verified the relevance of these genes to tumor biology using proof-of-concept siRNA-based experiments. We conclude that non-mutated genes are potentially important for ovarian cancer tumor biology and could lead to new therapeutic strategies.

## Methods

### In-silico mutagenesis approach

We obtained somatic mutation data from the TCGA Ovarian Cancer Project from the GDC Data Portal (https://portal.gdc.cancer.gov/). We implemented a method to efficiently simulate mutations across a set of nucleotide sequences in Matlab as previously described in Malek, Halabi and Rafii [13]. The TCGA-OV data consisted of 316 patients, so we performed a simulation run 316 times. Since mutations were random, each simulated run of 316 patients was also repeated 100 times. In total, there were 31,600 simulated runs. Each simulation run consisted of simulating the mutagenesis of 140,362,938 nucleotide bases. Furthermore, since different bases undergo different mutation rates, it was necessary to implement a way to differentially mutate different sets of nucleotide bases. The sequence space was therefore divided into nucleotide bases that were (1) A or T (2) C or G (3) CG or GC. Mutations at these different sets were assigned different mutation rates. We used the background mutation rates (BMR) published in the TCGA study [4] in Additional file 1: Table S2.2b (A/T mutations: 8.54 × 10–7, C/G mutations: 1.2 × 10–6, CG/GC mutations: 4.31 × 10–6 and insertions-deletions at 2.2 × 10–7).  Since no information about insertions-deletion sequence specificity was available, we added the indel mutation rate to the other categories. Three different random mutation vectors were generated of a length equal to the number of bases in the A/T, C/G, and CG/GC vectors. Each random mutation vector consisted of 0’s and 1’s with the frequency of 1’s occurring randomly at a density equal to the TCGA published background mutation rate. The three different mutation vectors were then combined to form the final mutation vector that had within it all simulated mutations. We used a reduced sequence library corresponding to the sequences that overlapped with the Agilent SureSelect v2 probe sequences. Obtaining the chromosomal locations of each probe from Agilent generated this reduced library. We then identified the regions corresponding to those probes by detecting the overlaps between the exon coordinates and the probe coordinates. The final exon sequence library consisted of 40,362,938 bases.  After carrying out simulated mutagenesis, the total number of mutations per gene was calculated by identifying all the exons corresponding to a gene. All exons sharing the same gene symbol were considered the same gene.

### Identification and pathway analysis of specific candidate genes

With the ability to calculate the simulated mutation frequency for each gene, it is possible to compare the observed mutation frequency in a gene with the expected simulated mutation frequency. To prioritize the genes with the largest deviations from random simulation expectation, we looked at the top 50 genes where the observed mutation rate was lower or higher than the expected mutation rate, comparing the observed and simulated frequencies based on rank of the observed/expected from simulation mutation frequency ratio. To guarantee coverage in the TCGA-OV dataset, we restricted our analysis to genes that were mutated at least once in the TCGA data since the publicly available data only included a list of mutations per patient and not the coverage across all positions.

We also performed pathway analysis using Ingenuity Pathway Analysis software (IPA from Qiagen, content version March 12, 2022). IPA software consists of a database of published relationships between genes with tools to analyze and visualize pathways. A list of the top 50 non-mutated genes with the observed/expected ratio of each gene was generated and uploaded to IPA. Network diagrams were generated among the genes in the list with genes colored in shades of red based on their observed/expected ratio with the reddest indicating the lowest ratio. Networks were either built using the IPA tools (CONNECT, PATHWAY EXPLORER, TRIM, KEEP) or identified automatically by IPA software as indicated. Automatically generated network significance was assessed with an IPA generated score which represents the negative exponent of the right-tailed Fisher’s exact test result (described in the IPA documentation: http://qiagen.secure.force.com/KnowledgeBase/articles/Basic_Technical_Q_A/Listing-of-Networks).

### Cell culture

We used three ovarian cancer cell lines for silencing experiments: SKOV3, OVCAR3 and APOCC. SKOV3 and OVCAR3 were purchased from ATCC and APOCC was derived in-house from ascites of a patient with Stage III serous adenocarcinoma. These cell lines were all maintained in DMEM high glucose (Hyclone, Thermo Scientific), 10% FBS (Hyclone, Thermo Scientific), 1% Penicillin-Streptomycin-Amphotericin B solution (Sigma), 1X Non Essential Amino-Acid (Hyclone, Thermo Scientific). Additionally, one non-cancer primary ovarian epithelial cell line was purchased from Sciencell (Cat. No. 7310) and cultured in poly-L-lysine-coated culture vessel (2 μg/cm2, T-75 flask) following ScienCell recommendations in Ovarian Epithelial Cell Medium (OEpiCM, Cat. No. 7311), 1% Ovarian Epithelial Cell Growth Supplement (OEpiCGS, Cat. No.7352) and 1% penicillin/streptomycin solution (p/s, Cat.No 0503). All cultures were incubated in humidified 5% CO2 incubators and the media was replaced every three days. For RNA sequencing, we used ovarian cancer cell lines SKOV3, APOCC, GOC-2, and GOC-A2 [14, 15], non-cancer ovary derived fibroblasts (ScienCell, Cat. No. 7330) and non-cancer primary ovarian epithelial cell lines (ScienCell, Cat. No. 7310). GOC-2 cells were isolated from a papillary serous ovarian cancer obtained after neoadjuvant chemotherapy while GOC-A2 were derived from a stage IIIc serous ovarian cancer [14]. GOC-2, GOC-A2 and fibroblasts were cultured in DMEM high glucose as previously described.

### Gene expression analysis of cell Lines and TCGA-OV patient data

RNA from six different cell lines were isolated using Qiagen Allprep DNA/RNA miniprep kit as per manufacturer instructions. Library preparation was done with Nugen’s Ovation Single Cell RNA-Seq System. Sequencing (Illumina 100 bp paired-end reads) was done on Illumina HiSeq 2500. Alignment was done with RNA Star to GRCH37 [16]. Mapping to genes was done with Rsubread using the FeatureCounts function [17]. Normalization and quantification of gene expression was done with edgeR [18]. All genes with any read count were included. The reads per kilobase of transcript per million mapped reads (RPKM) measure was calculated for all genes in all cell lines and used for distribution comparison.

Publicly available gene expression data from the TCGA-OV project was downloaded from the GDC data portal (https://portal.gdc.cancer.gov/legacy-archive) using the following filters: Primary-site = Ovary, Data-category = Gene expression and Platform = HT_HG-U133A. This data consisted of gene-level, robust multiarray analysis (RMA) normalized and background-corrected expression values for 12,042 genes from primary ovarian cancer biopsies. The RMA values were used as provided. The gene expression data files were further filtered to include only those that had somatic mutation data. Somatic mutation data was similarly obtained from the GDC data portal (https://portal.gdc.cancer.gov). The intersection between gene expression data and somatic mutation data files resulted in 315 samples for further expression analysis.

Custom scripts in Matlab software (Mathworks) were used for further analysis and visualization. To analyze the distribution of gene expression of both cell lines and TCGA-OV we used the non-parametric, two-sample Kolmogorov–Smirnov test as implemented in Matlab software (version 2019a).

### Methylation and copy number analysis of TCGA-OV primary ovarian cancer samples

We downloaded from the GDC data portal (data release 32) methylation Beta value data obtained from Illumina human methylation 27 chip from 605 samples. Beta values represent the fraction of methylation at a specific site with 0 representing no methylation and 1 representing complete methylation. We then excluded from the analysis non-primary and non-cancer samples which resulted in 582 samples for further analysis. We aggregated the Beta value data from all patients for the top 50 non-mutated (41 matches) and top 50 mutated genes (33 matches) and compared their distribution using the two-sample Kolmogorov–Smirnov test implemented in Matlab software (version 2021a). When one gene matched multiple methylation sites, the beta values were aggregated across the gene.

Similarly, for copy number analysis we downloaded from the GDC data portal (data release 32) ‘Gene Level Copy Number’ data obtained from the Affymetrix snp 6.0 array from 589 samples. We excluded non-primary cancer samples to obtain 562 samples for comparison. We matched 48 of the top 50 non-mutated genes and 43 of the top 50 mutated genes and aggregated all the copy number data across all samples. Distributions were compared using the non-parametric, two- sample Kolmogorov–Smirnov test as implemented in Matlab software (version 2021a).

### siRNAs screening system

Double-stranded siRNAs targeting each gene were obtained from Invitrogen (Silencer^®^ Select Pre-Designed siRNA LPP gene, Cat. No 4392420, siRNA ID: s8270, Silencer^®^ Select Pre-Designed siRNA TRAPPC9 gene, Cat. No 4392420, siRNA ID: s38115, Silencer^®^ Select Pre-Designed siRNA ELFN2 gene, Cat. No 4392420, siRNA ID: s41621, Silencer^®^ Select Pre-Designed siRNA ANKLE2 gene, Cat. No 4392420, siRNA ID: s23124, Silencer^®^ Select Pre-Designed siRNA PGR gene, Cat. No 4392420, siRNA ID: s10415, Silencer^®^ Select Pre-Designed siRNA MAP1B gene, Cat. No 4392420, siRNA ID: s8499, Silencer^®^ Select Pre-Designed siRNA VEGFA gene, Cat. No 4392420, siRNA ID: s461, Silencer^®^ Select Pre-Designed siRNA SLC12A9 gene, Cat. No 4392420, siRNA ID: s224445, Silencer^®^ Select Pre-Designed siRNA CELSR1 gene, Cat. No 4392420, siRNA ID: s18485). We also selected from Qiagen RNAi Human/Mouse starter kit Cat. No 301799, positive siRNA targeted against the protein kinase MAPK1, also called ERK2, and a non-targeting negative or non-silencing control siRNA that exhibits minimal nonspecific effects on gene expression and phenotype. Both the positive and negative controls were included in each 96-well plate.

To assess the degree of knockdown, cells were seeded in 96-well culture plates at a density of 5000 cells/well. cDNA synthesis was carried out 72 h after cell siRNA using TaqMan Gene Expression Cells-to-Ct kit (Thermo-Fisher). Normalization was done using the included B-actin probe in the Cells-to-Ct Control kit (Thermo-Fisher). All qPCR reactions were performed in triplicate and Cq values were averaged.

### Cell viability assay

We used Promega’s CellTiter-Glo^®^ assay in 96 well plates. Briefly, cells were seeded at 5000 cells per well in 96-well plates and allowed to attach overnight at 37 °C. Twenty-four hours after attachment, cells were transfected with individual siRNAs at 10 nM using Lipofectamine Max (Thermo Fisher). Twenty-four hours after siRNA treatment the transfection media was replaced with serum-free media. We used the same siRNA concentrations and transfection reagents in all cell lines and experiments. In addition, positive and negative siRNA controls were added in different wells. Seventy-two hours after transfection, 100 μl of CellTiter-Glo^®^ reagent was added to 100 μl of medium containing cells in a 96-well plate and viability was evaluated using EnVision Workstation version 1.12 from PerkinElmer. All experiments were performed in triplicates. Student’s t-test was used to compare the proliferation fraction of the knockdowns with that of the negative control. A p-value less than 0.05 was considered significant.

### Morphological marker staining

Cells were incubated 72 h after transfection with Invitrogen’s Live Cell stain CellMask Orange/Red for the plasma membrane and Hoechst 33342. Both the cell morphology of the cells and the nuclear morphology were visualized by confocal microscopy (Zeiss LSM 510).

### Wound healing assay

Cancer cells (50000 cells/well) were plated in 24-well plates in triplicate. Twenty-four hours after siRNA transfection, cells were starved from serum. A scratch was made in all the wells with a 1 μL pipette tip forty-eight hours after siRNA transfection. Images were taken directly after the scratch (0H) and again after 24 h (24H) and 48 h (48H). Edges were identified with manual inspection and wound healing was quantified as the ratio of the pixel distance at the timepoints relative to the 0H distance. Student’s t-test was used to calculate significance of differences between the ANKLE2 knockdown and the control by combining the 24H and 48H data.

### Chemotherapy

Paclitaxel/taxol and carboplatin were purchased from National Center for Cancer Care and Research (NCCCR; Doha, Qatar) pharmacy. Briefly, cancer cells (5000 cells/well) were plated in 96-well plates in triplicate for each condition. Twenty-four hours after siRNA treatment, the cells were starved from serum. Forty-eight hours after siRNA transfection, each drug suspended in phosphate buffered saline (PBS) was added to each well at a concentration of 50 µM and viability was analyzed after 24 h. Student’s t-test was used to compare the proliferation fraction of different pairs. A p-value less than 0.05 was considered significant.

## Results

### In-silico identification and pathway analysis of non-mutated genes

**Fig. 1:**
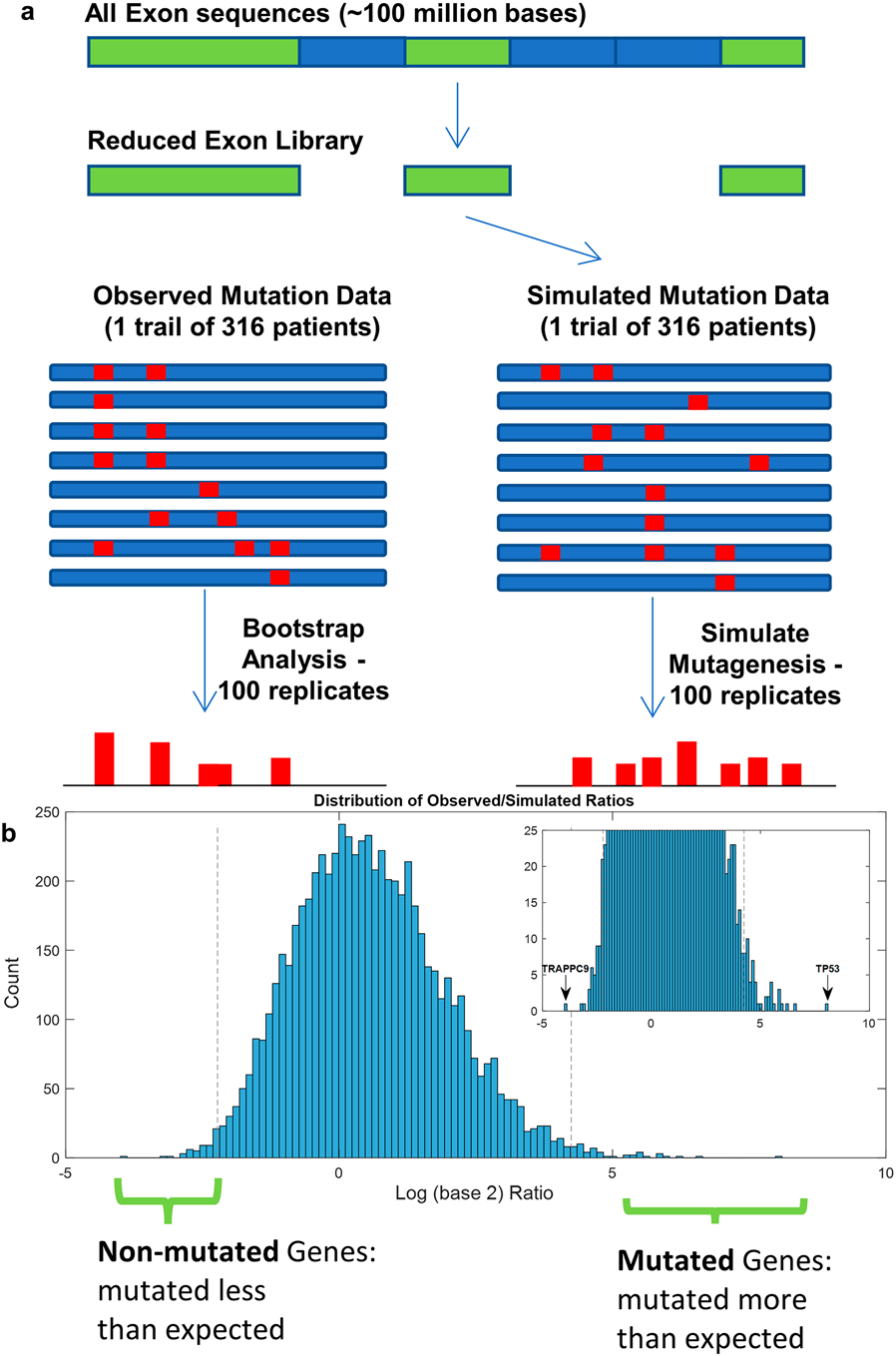
Identifying non-mutated genes. **a** Schematic of mutagenesis simulation. To approximate the data used in the patient exome sequencing, a reduced exon library was used consisting of the exons approximating those used in the TCGA trial. Simulated mutagenesis depicted as red lines is subjected to repeated trials using the observed background mutation frequencies. The mutation frequencies are then compared between the simulated and observed data. **b** Log ratio distribution of observed/simulated data. The inset limits the y-axis to 25 to see the distribution at the tails more clearly; the genes that are most extreme are labelled. Gray dashed lines show the top 50 non-mutated genes and top 50 mutated genes that are used for subsequent analysis (corresponding to the values less than 0.007 quantile and greater than the 99.23 quantile of the dataset)

We obtained the publicly available mutation data from the TCGA-OV project as described in the methods. The mutation data consisted of somatic mutations from ovarian cancer tissues in 316 patients. To determine which, if any, genes were potentially significantly non-mutated, we performed simulated mutagenesis on a reduced exon sequence library (Fig. 1a). We then compared the simulated mutagenesis results to the observed mutation data. Our comparison of simulated mutations to the observed mutations showed that most genes had a mutation rate similar to what is expected randomly, as the observed/simulated ratio was close to 1:1 for the vast majority of genes (Fig. 1b). However, a few genes were observed with mutatio n rates both higher and lower than expected (Fig. 1b, Table 1, Additional file 1: Table S1a). Among well-known genes, *TP53* and *MMP8* are mutated the most (Additional file 1: Table S1b). Notably, among the genes that mutated the least is vascular endothelial growth factor (*VEGFA*), a molecule that plays an established role in tumor angiogenesis [12].

**Table 1:** List of genes and known functions of the top 20 genes mutated less than expected

\	Gene name	Ratio	Obs. rank	Sim. rank	Annotation	Loc	Function	Refs
1	*TRAPPC9*	0.07	5891	2	trafficking protein particle complex 9	PM	NF-kappa-B signaling	[19]
2	*LPP*	0.11	3611	11	LIM domain containing preferred translocation partner in lipoma	NU	Localized at focal adhesions and cell–cell contact sites; shuttles to the nucleus where it affects transcription	[20–25]
3	*ELFN2*	0.12	4446	13	extracellular leucine-rich repeat and fibronectin type III domain containing 2	UK	Unknown	–
4	*ANKLE2*	0.13	3107	16	ankyrin repeat and LEM domain containing 2	NU	Mitosis	[26, 27]
5	*SHROOM3*	0.14	4931	28	shroom family member 3	CY	Regulating cell shape in certain tissues	[28–33]
6	*PGR*	0.14	3798	27	progesterone receptor	NU	Regulation of gene expression and affect cellular proliferation and differentiation in target tissues	[34–37]
7	*MAP1B*	0.15	6400	39	microtubule-associated protein 1B	CY	Microtubule assembly	[38–44]
8	*OBSL1*	0.15	5649	38	obscurin-like 1	PM	Links internal cytoskeleton to cell membrane	[45–47]
9	*CELSR1*	0.15	6483	57	cadherin, EGF LAG seven-pass G-type receptor 1	PM	Involved in cell adhesion	[48, 49]
10	*DBNL*	0.16	5544	50	drebrin-like	CY	For organ development and immune response. Promote proliferation, colony formation, migration, invasion	[50–53]
11	*PHF21A*	0.16	3552	36	PHD finger protein 21A	NU	Repression of neuron-specific genes through repressor element-1 or neural restrictive silencer	[54–56]
12	*TRPV6*	0.16	5245	48	transient receptor potential cation channel, subfamily V, member 6	PM	Calcium channels required for assembly and regulation	[57–63]
13	*VEGFA*	0.16	4406	47	vascular endothelial growth factor A	ES	Angiogenesis, endothelial cell growth, cell migration, apoptosis	[64–84]
14	*GRIP2*	0.17	3298	51	glutamate receptor interacting protein 2	OM	Bind AMPA receptors and target AMPA receptors to synapses	[85, 86]
15	*RALGDS*	0.17	4874	61	ral guanine nucleotide dissociation stimulator	CY	effectors of Ras-related GTPases that participate in signaling	[87, 88] [89]
16	*CCDC144A*	0.17	4302	58	coiled-coil domain containing 144A	UK	Unknown	–
17	*RAI1*	0.17	3399	55	retinoic acid induced 1	CY	associated with severity and response to medication in schizophrenia patients	[90–92]
18	*TBC1D2B*	0.17	3106	52	TBC1 domain family, member 2B	UK	Unknown	–
19	*SMG1*	0.17	5523	82	smg-1 homolog phosphatidylinositol 3-kinase-related kinase	CY	Involve in tumorigenesis as a new tumor suppressor	(93–95)
20	*BRWD1*	0.18	4873	85	bromodomain and WD repeat domain containing 1	NU	Unknown	–

Ratio: The ratio of the observed number of mutations to the simulated number of mutations. Obs. Rank: observed rank with 1 being the most mutated in the TCGA data. Sim. Rank: simulated rank with 1 being the most mutated in simulations.

Loc.: cellular localization, PM: Plasma membrane, NU: Nuclear, UK: Unknown, CY: Cytoplasm, ES: Extracellular space

We then conducted a detailed literature search on the top 20 genes mutated less than expected to understand if they could be playing an important role in tumor biology (Table 1). Although we found no relevant cancer literature for several of these genes, others were found to be highly interesting in terms of cell biology including *TRAPPC9*, which plays a role in NF-κB signaling, *ANKLE2*, which plays a role in mitosis, and *VEGFA*, which plays a role in angiogenesis (references provided in Table 1).

**Fig. 2:**
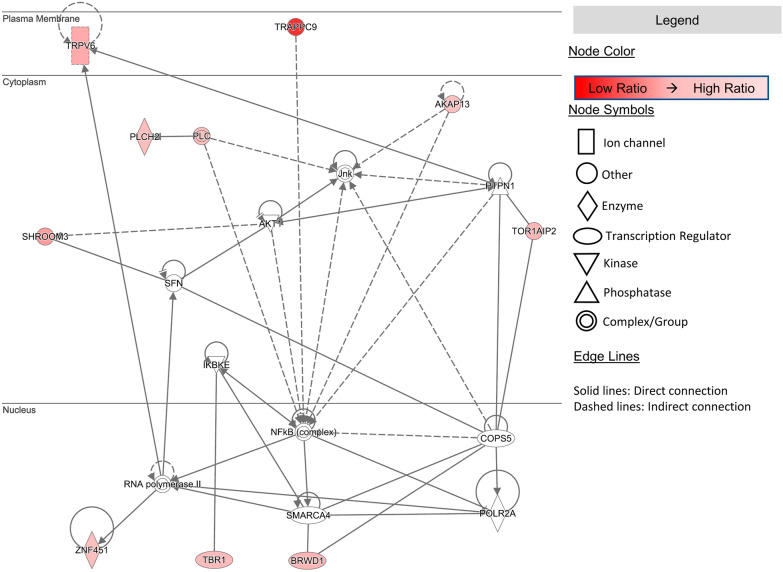
Pathway analysis of non-mutated genes. IPA generated pathway showing the interaction of genes within one network. The non-mutated gene with the highest difference from random expectation, *TRAPPC9*, is linked to a central NF-κB pathway along with other non-mutated genes. The shading as shown in the legend is proportional to the ratio of observed/simulated where genes that are redder have a lower ratio. Links between genes are either direct (**solid lines**) or indirect (**dashed lines**)

To understand further if these genes were acting independently or could be part of pathways, we performed network analysis on the set of 50 top non-mutated genes. We observed, with a constructed pathway, that ten of these genes interact directly or indirectly through the AKT and NF-κB pathways (Fig. 2). The activation of the AKT/ NF-κB pathways is associated with resistance to therapy in advanced ovarian cancer [14, 96]. IPA also automatically identified a set of genes that significantly interact with the NF-κB pathway shown as network 2 in Additional file 1: Figure S1a. Furthermore, three additional networks were automatically identified by IPA among the Top 50 non-mutated genes (Additional file 1: Figure S1a). We show one network consisting of 12 genes including ANKLE2, SHROOM3 and ELFN2 interacting through direct connections with VIRMA, a nuclear/cytosolic protein involved in RNA methylation/adenylation and implicated in different cancers [97, 98]. These results show that the identified non-mutated genes interact in cancer related biological pathways.

### Gene expression, methylation and copy number analysis of mutated and non-mutated genes

**Fig. 3:**
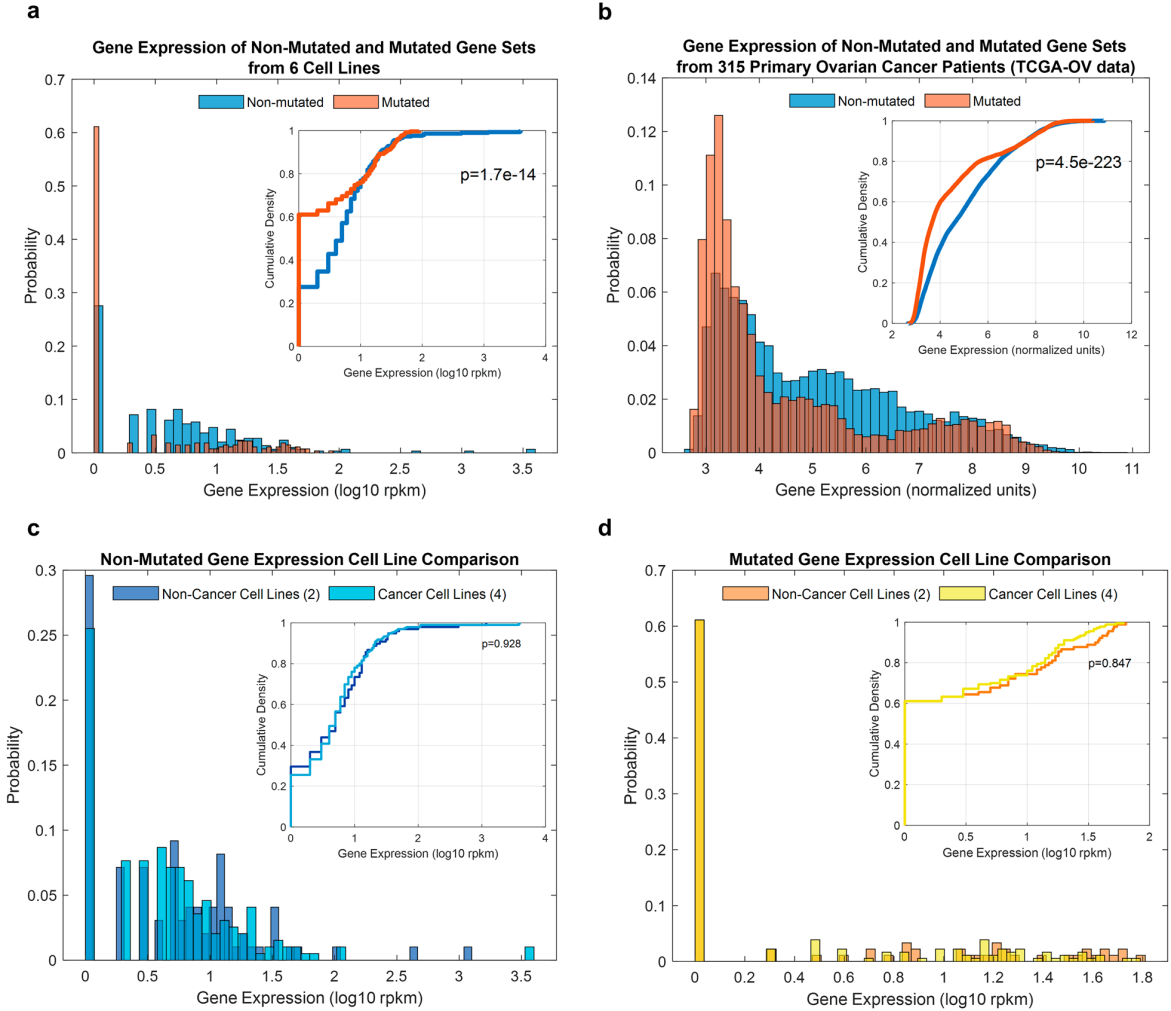
Gene expression distributions of non-mutated and mutated genes. Distribution of gene expression of non-mutated and mutated genes for our cell line data (**a**) and for patient data from TCGA-OV project (**b**). In blue is the expression distribution of top 50 non-mutated genes and in orange is the distribution of the top 50 mutated genes. Note that the expression of non-mutated genes is significantly higher than the expression of mutated genes in both the cell line and patient data. Distribution of gene expression of non-mutated genes (**c**) and mutated genes (**d**) in non-cancer cell lines and cancer lines. Note that there is no significant difference in gene expression difference between non-cancer and cancer cell lines in either mutated or non-mutated genes. The inset for each panel shows the cumulative density plots of the same data

To investigate further the biological relevance of our computational findings, we assayed the gene expression of multiple ovarian cancer and normal cell lines using RNA sequencing (Additional file 1: Figures S2 and S3). The set of genes that were mutated less than expected were found to be expressed at a higher level than genes mutated more than expected (Fig. 3a). We confirmed similar findings using the TCGA-OV patient data (Fig. 3b) which consists of gene expression data from 315 ovarian cancer biopsies from 315 different patients. To determine if the mutation itself can affect gene expression we looked at expression distributions when a gene is both mutated and non-mutated in the TCGA-OV data and no significant difference was observed between mutation state and expression state in 25 out of 28 genes (Additional file 1: Figure S4). The best example of this is seen in the TP53 gene which has the most mutations; the expression level of the mutated and non-mutated TP53 samples have a similar distribution (Additional file 1: Figure S4).

We also compared the expression of non-mutated genes between cancer cell lines and normal cell lines (Fig. 3c) and the expression of mutated genes between cancer cell lines and normal cell lines (Fig. 3d). We found no significant difference in either of these comparisons in contrast to the significant differences across the gene sets. Moreover, we searched across the top 50 non-mutated genes shown in Additional file 1: Figure S2 for genes whose expression is low in non-cancer cells and high across all cancer cells. We could not identify such genes but we did identify several non-mutated genes whose expression was low in both non-cancer cell lines and high in three out of the four cancer cell lines. These genes include ELFN2, CELSR1 and TRPV6. We therefore conclude that non-mutated genes could play a role in tumor biology due to their relatively high expression when compared to the expression of the most mutated genes.

We then performed an analysis of methylation states comparing the aggregated methylation beta value of non-mutated and mutated genes using the TCGA-OV data as described in the Methods. As shown in Additional file 1: Figure S5a, non-mutated genes are overall significantly less methylated than mutated genes. Non-mutated genes show higher peaks than mutated genes at low beta values (0 to 0.2) while mutated genes show higher peaks than non-mutated genes at high beta values (0.8–1). Following the methylation analysis, we also performed copy-number analysis on the same TCGA-OV dataset as shown in Additional file 1: Figure S5b. Here, we also find a significant difference between non-mutated and mutated genes with non-mutated genes having overall more genes with copy number greater than 2 while mutated genes having more genes with copy number less than 2. The methylation and copy number results are consistent with the gene expression results. Lower methylation and higher copy number is associated with greater gene expression which is what we observe when comparing non-mutated genes to mutated genes.

### Functional effect of siRNA knockdown of non-mutated genes

**Fig. 4:**
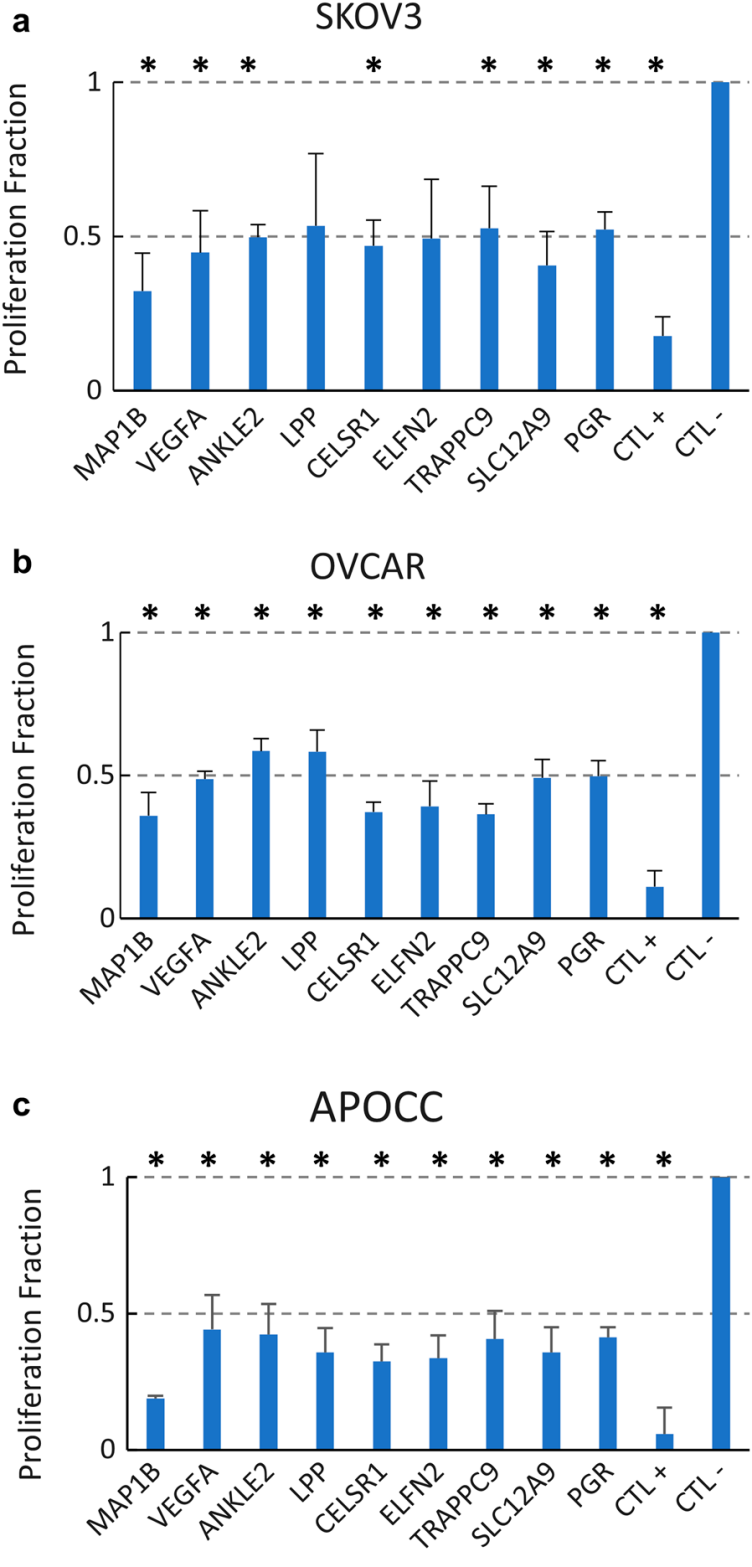
Cell proliferation after selected gene knockdown in three different ovarian cancer cell lines: SKOV3 (**a**), OVCAR (**b**) and APOCC (**c**). Fraction of cell viability is standardized against the negative control (CTL-) proliferation levels and the error bars are the standard deviations from three replicates. A reference line is drawn through 0.5 showing that almost all genes in all tested cell lines reduced cell proliferation by at least 50%. Asterisks denote significant differences (p-value < 0. 05) between the gene knockdown and the negative control

To determine if non-mutated genes could directly impact cancer cell line growth we selected nine genes for proof-of-concept in-vitro gene silencing experiments. We selected seven genes among the top 10 ranked genes (rank 1–4, 6–7, and 9), *VEGFA* (rank 14), as it is known to be involved in angiogenesis, and *SLC12A9* (rank 41), as it is a plasma membrane-embedded cation transporter which may be more easily targeted. We first determined that the transcripts were successfully knocked down at levels greater or equal to 50 percent of the negative control (Additional file 1: Figure S7). We then performed silencing experiments on these nine genes with three different ovarian cancer cell lines (Fig. 4) and one non-cancer ovarian epithelial cell line (Additional file 1: Figure S6). We found a significant reduction of viability following silencing of 7 out of 9 genes in SKOV3, 9 out of 9 genes in OVCAR, 9 out of 9 genes in APOCC and 2 out of 9 genes in the non-cancer ovarian epithelial cell line. We therefore conclude that silencing of these genes affects cancer cell lines significantly more than the non-cancer cell line.

**Fig. 5:**
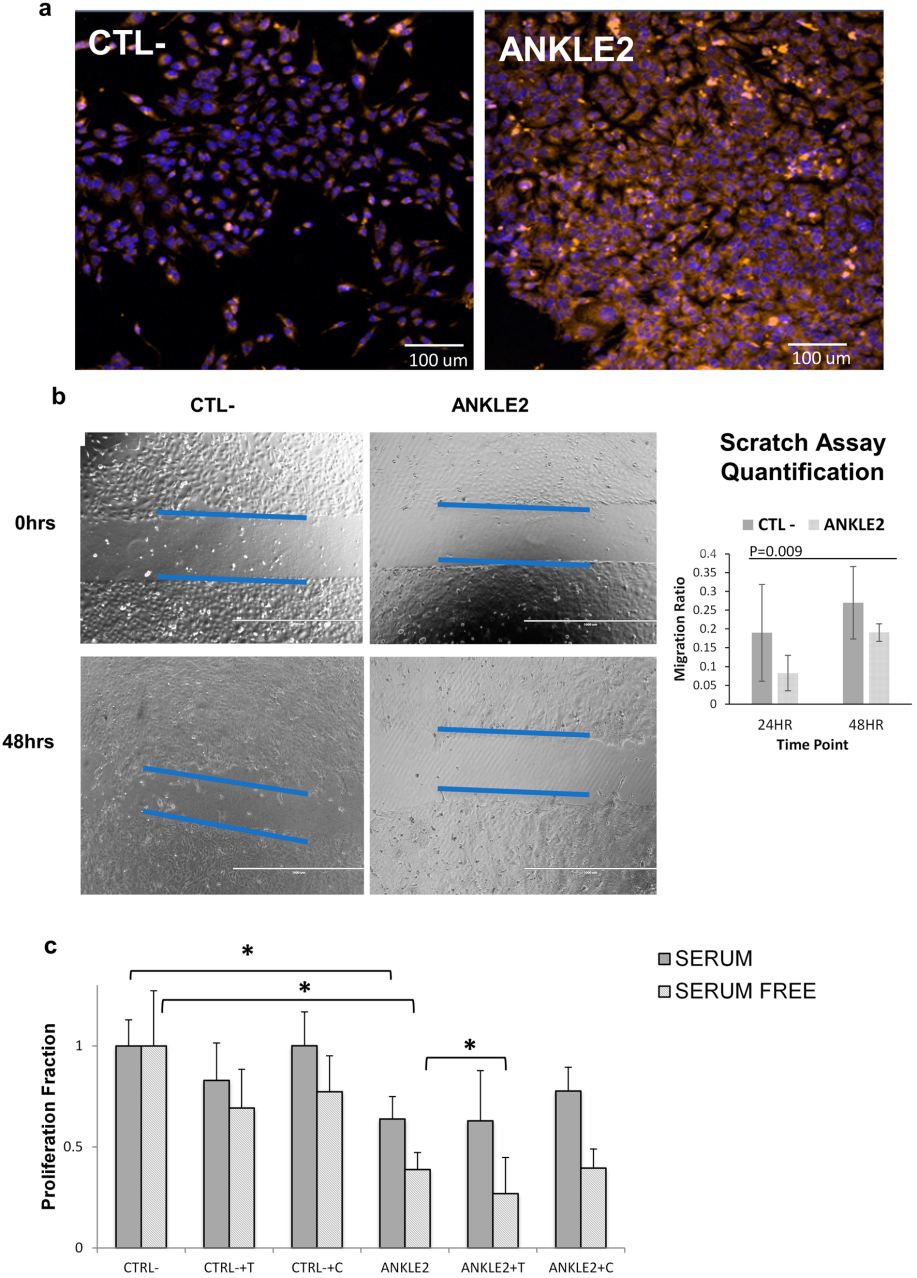
ANKLE2 knockdown in SKOV3 cell line. **a** Representative fluorescent images of microscopic imaging of SKOV3 after *ANKLE2* and CTL- knockdowns. **b** Migration assay for SKOV3 after knockdown with selected genes. Images at the top were taken just after the scratch while the lower images were taken after 48 h. The blue lines denote the margins of the scratch. The white horizontal line is 200 µm. The chart to the right shows quantification of the migration normalized to the negative control at two different time points. The p-value (P) of a pairwise t-test combining the differences at the 24HR and 48HR time points is shown in the chart. **c** Chemosensitivity of CTL- (negative control) and ANKLE2 knockdown in SKOV3 cells with and without paclitaxel/taxol (T) and carboplatin (C) in serum containing and serum free media. Errors bars are the standard error of the mean of three replicates. Values are normalized to the negative control (CTL-). Asterisks denote significant differences between the indicated pairs

To examine further functional effects, we selected *ANKLE2* as it was interestingly found to play a role in cell division [26, 99, 100]. Silencing of *ANKLE2* resulted in significant morphologic changes in SKOV3 (Fig. 5a) where cells were growing in packed scattered colonies with a fibroblast-like shape before the knockdown. We also observed a significant reduction in migration in SKOV3^ANKLE2−SiRNA^ using a scratch assay (Fig. 5b). Finally, we evaluated the impact of *ANKLE2* knockdown on chemoresistance. SKOV3 cells are paclitaxel/taxol-resistant [101, 102] but we found that *ANKLE2* knockdown enhanced the cytotoxic effects of paclitaxel compared with negative controls as shown in Fig. 5c. In contrast to paclitaxel effects, no chemotherapeutic sensitivity was observed with carboplatin (Fig. 5c).

## Discussion

Here we focused on better understanding the role of non-mutated genes in ovarian cancer. We showed that in a few genes there are differences between simulated and observed mutation frequencies in the TCGA ovarian cancer cohort of 316 patients. These genes fell into two categories—genes that are mutated more than expected (such as *TP53*) and genes that are mutated less than expected which we call here non-mutated genes. The non-mutated gene set was especially interesting because this was a set of genes that could be selected against mutation due to their role in tumor biology and that could offer new therapeutic strategies. The TCGA study in ovarian cancer uncovered 9,984 genes mutated in 316 patients with a very heterogeneous distribution among patients [94]. Not only are there many mutations but, with the exception of *TP53*, patients share few mutations. This mutational diversity makes treatment strategies that target mutated genes difficult as every patient may have a different combination of mutated genes. However, treatment strategies that target non-mutated genes may be more effective as these non-mutated genes would be the same in different patients if the observed non-mutation is due to selection against mutation. Indeed, one of the genes we identified as non-mutated is *VEGFA*, which is known to be involved in promoting cancer angiogenesis [65–67, 69, 103].

We found that the non-mutated genes are members of cancer-relevant networks. For example, *SHROOM3* has a role in regulating cell shape in tissues [30] and is connected with the SNF complex, which mobilizes nucleosomes, remodels chromatin and opens up the transcription-binding domains leading to an increase in transcription [104]. Growing studies support the role of SNF complex in cancer development, as several subunits possess intrinsic tumor-suppressor activity [105]. Furthermore, several non-mutated genes interact indirectly and directly with the AKT network which modulates the function of numerous substrates involved in the regulation of cell survival, cell cycle progression and cellular growth, and neo-vascularization [106, 107]. One of the most interesting genes we observed to be significantly non-mutated was *ANKLE2*, which is a member of the LEM family of inner nuclear membrane proteins. This gene functions as a mitotic regulator through the post-mitotic formation of the nuclear envelope [26]. Our inhibition strategy confirmed the important role of *ANLKE2* in different tumor-associated phenotypic traits.

We observed that generally non-mutated genes are well-expressed in both non-cancer and cancer tissues. This could limit the clinical use of targeting non-mutated genes as there could be significant side effects due to deleterious effects on non-cancer cells. However, targeting non-mutated genes could still be a viable strategy if cancer cells display greater sensitivity than non-cancer cells to inhibition of non-mutated genes. The greater sensitivity of cancer cells to radiation or chemotherapy compared to non-cancer cells has resulted in the wide use of these treatment modalities although with significant side effects. We have performed one experiment showing that non-cancer ovarian epithelial cells are less susceptible than cancer cell lines to the effects of silencing in our viability assay. While these results are promising, they need to be further validated across different cell lines and esp ecially across different cellular contexts. Cells grown in 2D monocultures are very different from cells grown in co-culture with other cells or in 3D organoids and from cells in tissues. It will be interesting to explore the differential sensitivity of cancer cells and non-cancer cells to non-mutated gene inhibition in future studies. Furthermore, we identified several non-mutated genes where three out of four cancer cells had high expression but where expression was low in non-cancer cells. These genes may be interesting therapeutic targets if this pattern is also seen across more cancer and non-cancer cells as targeting them could result in reduced toxicity t o non-cancer cells.

A related point that could affect therapeutic effectiveness is if these genes might be non-mutated because they are housekeeping genes and any mutation would be highly deleterious to all cells. The commonly known housekeeping genes are the *ACTB* gene, which is part of actin protein family, *RAB7A*, which belongs to the RAS oncogene family, and the *GAPDH* gene (Glyceraldehyde 3-phosphate dehydrogenase). In our study, they did not display any selection against mutation. The top 50 non-mutated genes identified are not part of classical housekeeping genes to our knowledge.

Our analysis is novel, as most studies have focused on mutated genes. Further data can help refine our analysis, as we used only the restricted publicly available datasets in this work. Sequencing with better coverage, such as whole-genome sequencing, would be an improvement to this analysis since we would get a much better coverage distribution. In addition, it would be interesting in future studies to develop single-cell and deep sequencing experiments in rapidly div iding cancer cells in culture across different time points to determine the distribution of mutations including low frequency mutations. With sufficient coverage it will also be possible to determine if there are significantly non-mutated genes in this context.

Comparing our data to the TCGA-OV study [4] shows that among the top 50 mutated genes we identified are two of the nine genes the TCGA identified as being significant. These genes are TP53 and RB1. The TCGA-OV used complex statistical models considering sequence context in addition to considering the ove rall prevalence of mutations. Our random mutation model here is relatively simple and the mutation probability of a specific base is independent of any other base. One possibility this limitation raises is that genes can be observed to be non-mutated not because they are selected against but because they have a sequence context that greatly reduces the chance of mutation. While these mutation-resistant genes could still make interesting targets if cancer cells are sensitive to them, their identification would require both high coverage data and improved mutation simulation models. Our overall approach here was to combine random simulation results with pathway analysis, gene expression and functional testing of selected genes.

In this study, we exploited large-scale cancer genomic databases and bioinformatics approaches to discover novel therapeutic candidates. Our combined bioinformatics and silencing approach could potentially lead to discoveries of interesting candidates without the need for complex, costly, high-throughput screening approaches. Understanding the broader landscape of non-mutated genes using combined TCGA datasets could lead to understanding key selection processes in place in cancer evolution and identifying critical steps that could be used as therapeutic targets.

## Conclusions

While extensive cancer research has focused on understanding genes whose mutations are selected for (mutated genes), comparatively little is known about genes whose mutation is selected against (non-mutated genes). Identifying non-mutated genes could lead to new therapies as non-mutated genes could be important for cancer survival and growth. We first identified potential non-mutated genes by comparing mutations observed in an ovarian cancer cohort with mutations expected in a random mutagenesis model and selecting genes with the greatest difference from random expectation. We then found that non-mutated genes interact in known pathways and are well-expressed in cell lines and patient tumors suggesting functional importance. Finally, we found that when we reduced the expression of selected non-mutated genes in ovarian cancer cell lines, the growth of all the cell lines was significantly reduced. This study is a first proof-of-concept showing that targeting non-mutated genes is a plausible cancer therapy approach.

## Supplementary Information

**Additional file 1: Table S1.** Top 20 genes mutated more and less than random expectation; **Figure S1.** Automated pathway analysis of non-mutated genes; **Figure S2.** Expression of top 50 genes mutated less than expected in different cell lines; **Figure S3.** Expression of top 50 genes mutated more than expected in different cell lines; **Figure S4.** Gene-level expression distribution in TCGA-OV patients in mutated and non-mutated samples; **Figure S5.** Methylation and copy number analysis in TCGA-OV data; **Figure S6.** Cell viability in non-cancer ovarian epithelial cells; **Figure S7.** Knockdown efficiency.

## Data Availability

Cell line RNA sequencing data used for gene expression analysis is available at the Gene Expression Omnibus repository with accession codes GSE75935 and GSE202628.

## Abbreviations

HGS-OvC: High Grade Serous Ovarian Carcinoma
TCGA: The Cancer Genome Atlas
BMR: Background Mutation Rate
