# Supplementary material for: Discovery of new therapeutic targets in ovarian cancer through identifying significantly non-mutated genes
Source: J Transl Med. 2022 May 26;20:244. doi: 10.1186/s12967-022-03440-5 (PMC9134657; doi:10.1186/s12967-022-03440-5)
Supplement: Supplementary file 1 — Additional file 1: Table S1. Top 20 genes mutated more and less than random expectation; Figure S1. Automated pathway analysis of non-mutated genes; Figure S2. Expression of top 50 genes mutated less than expected in different cell lines; Figure S3. Expression of top 50 genes mutated more than expected in different cell lines; Figure S4. Gene-level expression distribution in TCGA-OV patients in mutated and non-mutated samples; Figure S5. Methylation and copy number analysis in TCGA-OV data; Figure S6. Cell viability in non-cancer ovarian epithelial cells; Figure S7. Knockdown efficiency. [file 12967_2022_3440_MOESM1_ESM.pdf]

a

TOP 20 GENES WITH OBSERVED MUTATION FREQUENCIES LOWER THAN RANDOM EXPECTATION					
	Gene Name	Ratio Observed/Simulated	Observed Rank	Simulated Rank	Annotation
1	TRAPPC9	0.07	5891	2	trafficking protein particle complex 9
2	LPP	0.11	3611	11	LIM domain containing preferred translocation partner in lipoma
3	ELFN2	0.12	4446	13	extracellular leucine-rich repeat and fibronectin type III domain containing 2
4	ANKLE2	0.13	3107	16	ankyrin repeat and LEM domain containing 2
5	SHROOM3	0.14	4931	28	shroom family member 3
6	PGR	0.14	3798	27	progesterone receptor
7	MAP1B	0.15	6400	39	microtubule-associated protein 1B
8	OBSL1	0.15	5649	38	obscurin-like 1
9	CELSR1	0.15	6483	57	cadherin, EGF LAG seven-pass G-type receptor 1 (flamingo homolog, Drosophila)
10	DBNL	0.16	5544	50	drebrin-like
11	PHF21A	0.16	3552	36	PHD finger protein 21A
12	TRPV6	0.16	5245	48	transient receptor potential cation channel, subfamily V, member 6
13	VEGFA	0.16	4406	47	vascular endothelial growth factor A
14	GRIP2	0.17	3298	51	glutamate receptor interacting protein 2
15	RALGDS	0.17	4874	61	ral guanine nucleotide dissociation stimulator
16	CCDC144A	0.17	4302	58	coiled-coil domain containing 144A
17	RAI1	0.17	3399	55	retinoic acid induced 1
18	TBC1D2B	0.17	3106	52	TBC1 domain family, member 2B
19	SMG1	0.17	5523	82	smg-1 homolog, phosphatidylinositol 3-kinase-related kinase (C. elegans)
20	BRWD1	0.18	4873	85	bromodomain and WD repeat domain containing 1

b

TOP 20 GENES WITH OBSERVED MUTATION FREQUENCIES HIGHER THAN RANDOM EXPECTATION					
#	Gene Name	Ratio Observed/Simulated	Observed Rank	Simulated Rank	Annotation
1	TP53	271.9	1	3426	p53 tumor suppressor
2	MMP8	93.3	408	6505	matrix metallopeptidase 8
3	ANO4	76.5	509	6501	anoctamin 4, may act as a calcium-activated chloride channel
4	PAK3	65.4	537	6493	p21 protein (Cdc42/Rac)-activated kinase 3
5	SPINK5	58.6	1632	6507	serine peptidase inhibitor, Kazal type 5
6	SDC2	56.6	2490	6508	cell surface-associated heparan sulfate proteoglycan 1
7	MYBPC2	56.3	652	6479	myosin binding protein C, fast type
8	OVCH1	47.3	338	6425	ovochymase 1
9	MST4	46.8	1663	6504	serine/threonine protein kinase, may function in apoptosis
10	PNLIP	45.6	2301	6502	pancreatic lipase
11	ACADSB	44.9	2676	6506	acyl-CoA dehydrogenase, short/branched chain
12	NCAM2	44.2	708	6459	neural cell adhesion molecule 2
13	ALDH1A1	42.7	1123	6491	aldehyde dehydrogenase 1
14	ATP8A2	41.8	484	6451	ATPase, aminophospholipid transporter, class I, type 8A, member 2
15	EFR3A	38.9	796	6471	EFR3 homolog A, function not well-defined
16	UBLCP1	38.4	4365	6509	ubiquitin-like domain containing CTD phosphatase 1
17	CELA2A	32.1	2640	6497	chymotrypsin-like elastase family, member 2A
18	ANXA7	29.1	1771	6490	annexin-7
19	LTV1	28.4	2356	6482	LTV1 homolog, involved in export of ribosomal subunits
20	IFLTD1	28.4	2390	6485	intermediate filament tail domain containing 1

Table S1: Top 20 genes mutated less (a) and more (b) than random expectation by ratio of observed/simulated**.** The ratio is calculated as the average of multiple simulation runs. The rank is from the most mutated to the least mutated. For example, TP53 is the most frequently mutated gene in observed data while TRAPPC9 is the second most frequently mutated in the simulated data. VEGFA is in green font and TP53 is in red font.

a

ID	Molecules in Network	Score	Focus Molecules	Top Diseases and Functions
1	Actin, ↓**AKAP13,** Akt, ↓**BCR,** CG, Collagen type I (complex), Creb, Cyclin A, ↓**EEF1D,** ↓**EIF3B,** estrogen receptor, Fibrinogen, ↓**FN1,** FSH, ↓**GRIP2,** Hif1, ↓**ICE2,** Ige, ↓**MAP1B,** Pdgf (complex), ↓**PGR,** ↓**PHF21A,** Pka, PLC, ↓**PLCH2,** ↓**PTPRB,** Rac, ↓**RALGDS,** Rap1, RNA polymerase II, RPS6KA, ↓**SLC12A1,** ↓**SMG1,** ↓**TRPV6,** ↓**VEGFA**	40	17	Cancer, Organismal Injury and Abnormalities, Skeletal and Muscular Disorders
2	Ap1, ↓**ARHGAP29,** ↓**ARL10,** ↓**ATXN2,** ↓**BAHCC1,** CD3, ↓**DBNL,** ERK1/2, F Actin, Focal adhesion kinase, Histone h2a, Histone h3, Histone h4, Hsp90, Immunoglobulin, Insulin, Jnk, ↓**KDM2B,** ↓**LIMD1,** ↓**LPP,** ↓**MACROH2A1,** Mapk, Mek, NFkB (complex), P38 MAPK, PI3K (complex), Pkc(s), RAS, Ras homolog, Sfk, ↓**SHROOM3,** SRC (family), ↓**TRAPPC9,** Vegf, ↓**ZNF451**	26	12	Cellular Assembly and Organization, Connective Tissue Disorders, Developmental Disorder
3	↓**ANKLE2,** ANKRD12, BSG, ↓**BTBD7,** ↓**CCDC144A,** CTDSPL2, ↓**ELFN2,** FAM162A, GRAMD1B, GREB1, KANK4, MAML3, ↓**MCM9,** miR-12183-5p (and other miRNAs w/seed CUUCUUC), miR-324-3p (miRNAs w/seed CCACUGC), miR-6801-3p (and other miRNAs w/seed CCCCUGC), NCOA3, NUDT11, PRAG1, RAB11FIP4, ↓**RAI1,** RFXANK, ↓**SHROOM3,** ↓**SLC12A9,** SLC45A4, SNX16, TBC1D1, ↓**TBC1D2B,** ↓**TBR1,** ↓**TTLL3,** URB2, UTP20, VIRMA, ZFYVE9, ↓**ZNF646**	26	12	Cancer, Organismal Injury and Abnormalities, Renal and Urological Disease
4	7S NGF, ↓**BRWD1,** ↓**CELSR1,** ↓**CHD2,** DHX29, DNAAF1, ESR1, GGNBP2, H19, H3C13, H4C2, HDAC1, HOTAIR, ↓**L3MBTL1,** Macf1, ↓**MAP3K9,** mir-140, mir-322, MYC, NR3C1, ↓**OBSL1,** POU5F1, PRDM11, ↓**RBM33,** ↓**SHROOM3,** SON, TESMIN, TFB1M, Top2, ↓**TOR1AIP2,** TP53, TRAP/Media, ↓**WDR33,** ↓**WDR6,** WDR75	23	11	Cell Cycle, Cell Morphology, Hair and Skin Development and Function

b Pathway diagram of network 3

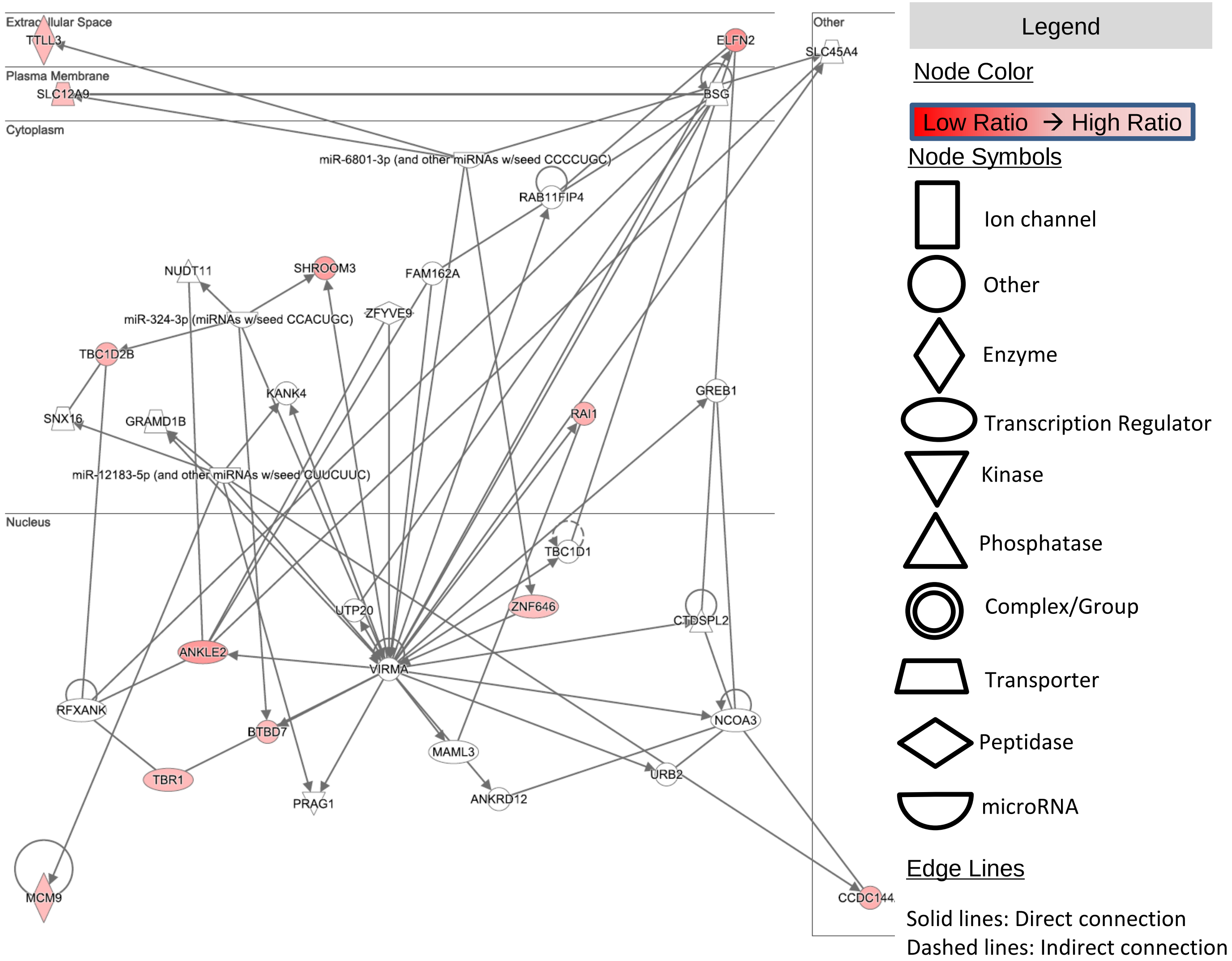

Figure S1: Automatically Generated IPA Pathways of the top 50 non-mutated genes. a) Four networks with IDs 1 to 4 (column 1) sorted by score were generated by IPA consisting of non-mutated genes and additional genes. The molecules in the network are provided in column 2 with the non-mutated genes indicated in BOLD with a downward pointing red arrow. The significance score is shown in column 3. The number of non-mutated genes in each pathway are in column 4 (Focus molecules). The IPA designated disease or function is shown in column 5. b) IPA generated visualization of network 3 containing ANKLE2 and other non-mutated genes.

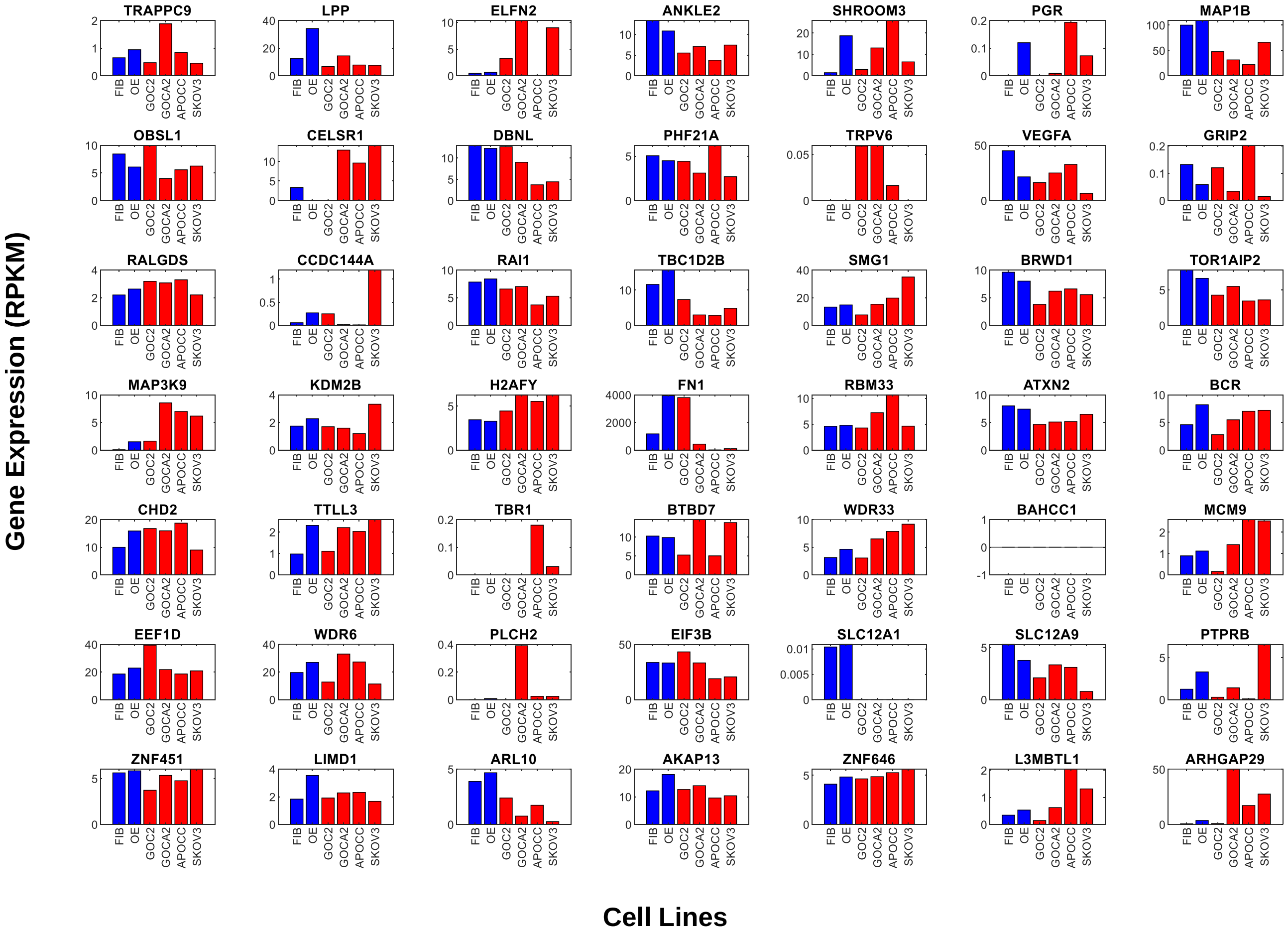

Figure S2 : Expression of top 50 genes mutated less than expected in different cell lines. The x-axis for each bar plot shows the cells lines and the y-axis show the gene-length normalized expression values. A line through 0 indicates no detectable expression for any cell line. The non-cancer cells lines are colored blue and are OE (ovarian epithelial) and FIB (fibroblasts) The ovarian cancer cell lines are colored red and are GOC2, GOCA2, APOCC and SKOV3. Gene names of 49 of the top 50 non-mutated genes were found in the RNA-sequencing data.

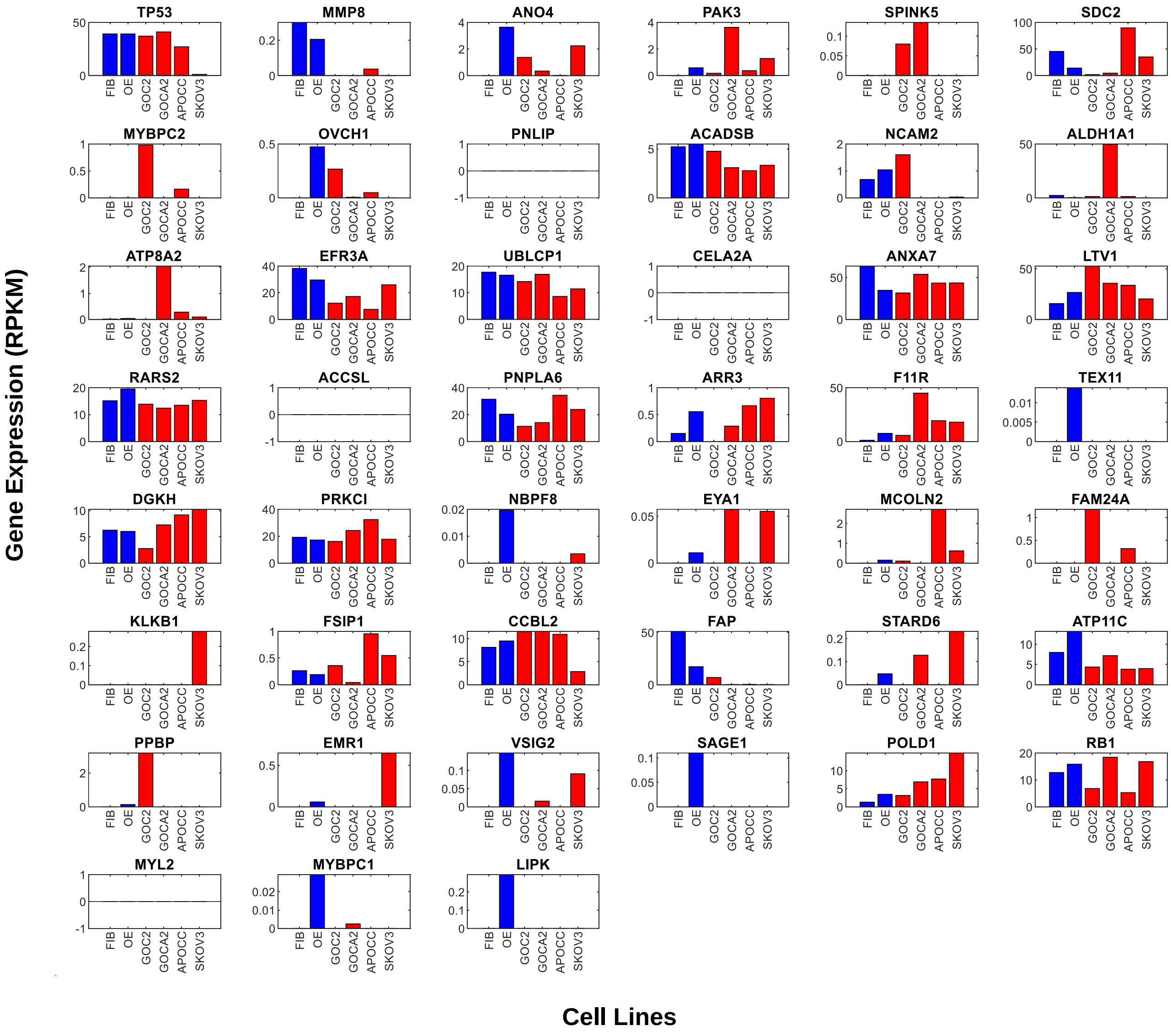

Figure S3: Expression of top 50 genes mutated more than expected in different cell lines. The x-axis for each bar plot shows the cells lines and the y-axis show the gene-length normalized expression values. A line through 0 indicates no detectable expression for any cell line. The non-cancer cells lines are colored blue and are OE (ovarian epithelial) and FIB (fibroblasts) The ovarian cancer cell lines are colored red and are GOC2, GOCA2, APOCC and SKOV3. Gene names of 45 of the top 50 mutated genes were found in the RNA-sequencing data.

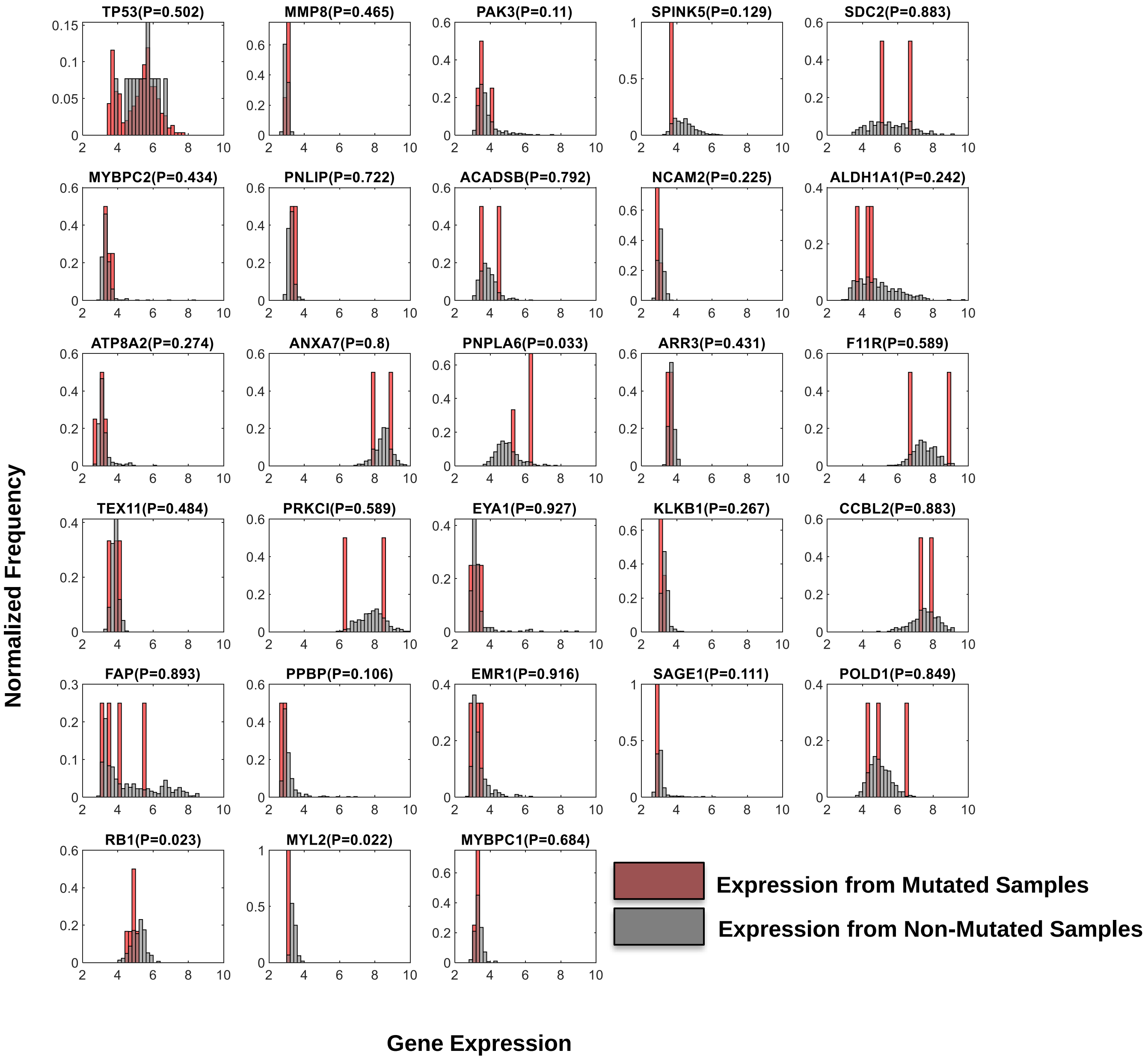

Figure S4: Gene-level expression distribution in TCGA-OV patients in mutated and non-mutated samples. This data is based on the TCGA data where both expression and mutation data is available for different genes. The x-axis for each histogram shows the gene expression from microarray data and the y-axis the frequency normalized to 1. The title shows the gene name and the p-value (P) from a two-sample Kolmogorov-Smirnov test of the expression values of mutated and non-mutated samples for the same gene.

a

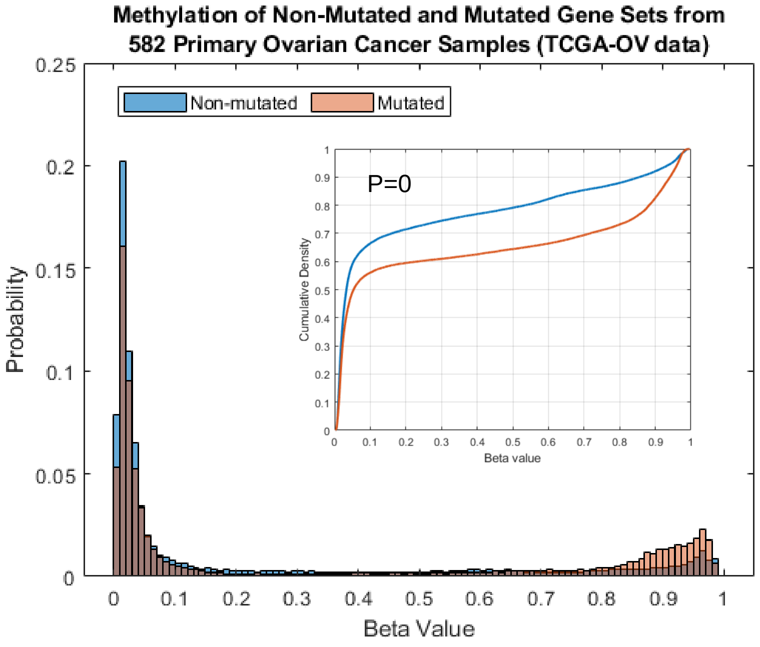

b

Non-mutated		
Value	Count	Percent
0	4	0.01%
1	2975	11.03%
2	10231	37.93%
3	6202	22.99%
4	4597	17.04%
5	1424	5.28%
6	902	3.34%
7	293	1.09%
8	165	0.61%
9	65	0.24%
10	45	0.17%
11	31	0.11%
12	15	0.06%
13	6	0.02%
14	1	0.00%
15	5	0.02%
16	1	0.00%
17	1	0.00%
19	3	0.01%
23	2	0.01%
28	1	0.00%
29	1	0.00%
31	1	0.00%
32	1	0.00%
35	1	0.00%
36	1	0.00%
Sum:	26974	

Mutated		
Value	Count	Percent
0	23	0.10%
1	3304	13.67%
2	9252	38.29%
3	5385	22.28%
4	3863	15.99%
5	1126	4.66%
6	658	2.72%
7	222	0.92%
8	134	0.55%
9	92	0.38%
10	36	0.15%
11	27	0.11%
12	13	0.05%
13	8	0.03%
14	5	0.02%
15	2	0.01%
16	4	0.02%
17	4	0.02%
18	1	0.00%
19	1	0.00%
20	1	0.00%
21	1	0.00%
22	1	0.00%
23	1	0.00%
27	1	0.00%
67	1	0.00%
Sum:	24166	

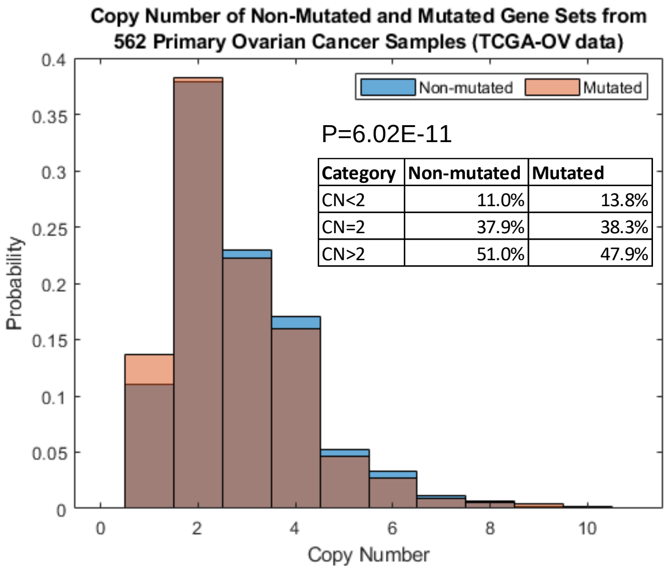

Figure S5: Methylation and copy number changes in primary ovarian tumor samples from the TCGA-OV project. a) Methylation beta value distributions in non-mutated (blue) and mutated (orange) samples. The inset shows the cumulative density plot of the same data with the p-value shown as P. In this case the p-value was 0 due to reaching the Matlab calculation limit. b) Aggregated copy number states are shown in the tables on the left for non-mutated and mutated genes and as a histogram on the right (limited at copy number =10). The histogram inset further shows copy number (CN) bins (CN less than 2, CN=2 and CN>2). The p-value is shown as P in the histogram on the right.

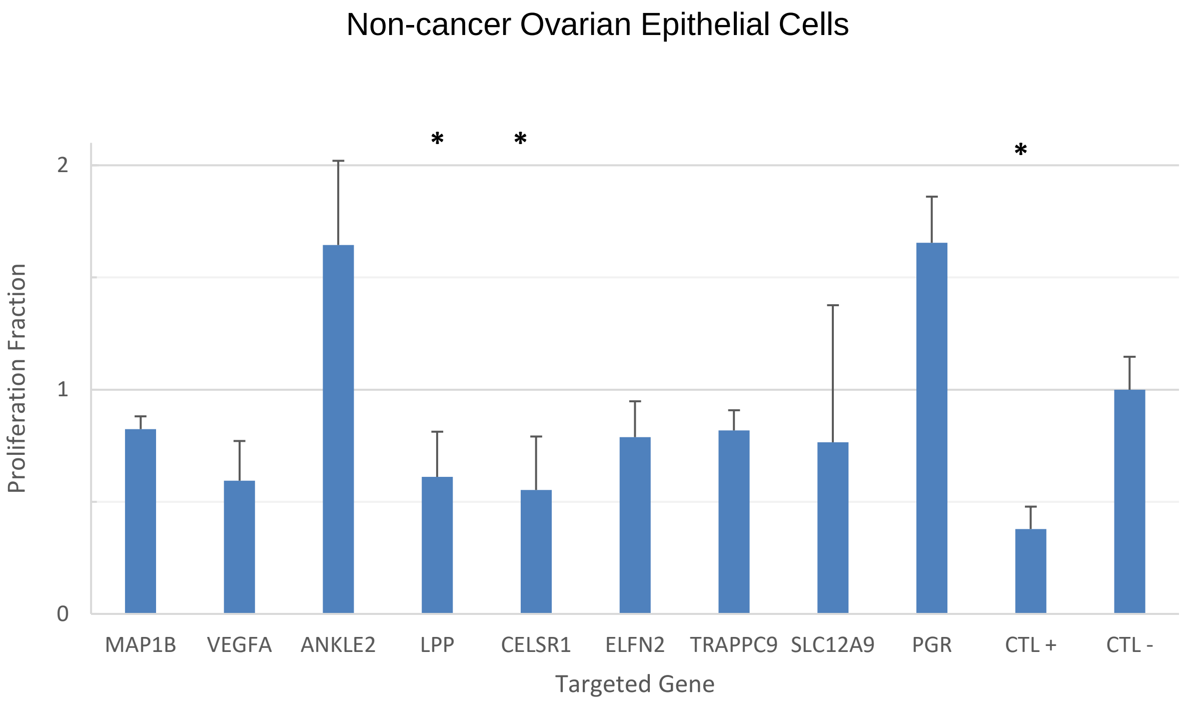

Figure S6: Cell viability relative to negative control of non-cancer ovarian epithelial cells. Asterisks denote significant differences between the targeted gene and the negative control (p-value < 0.05).

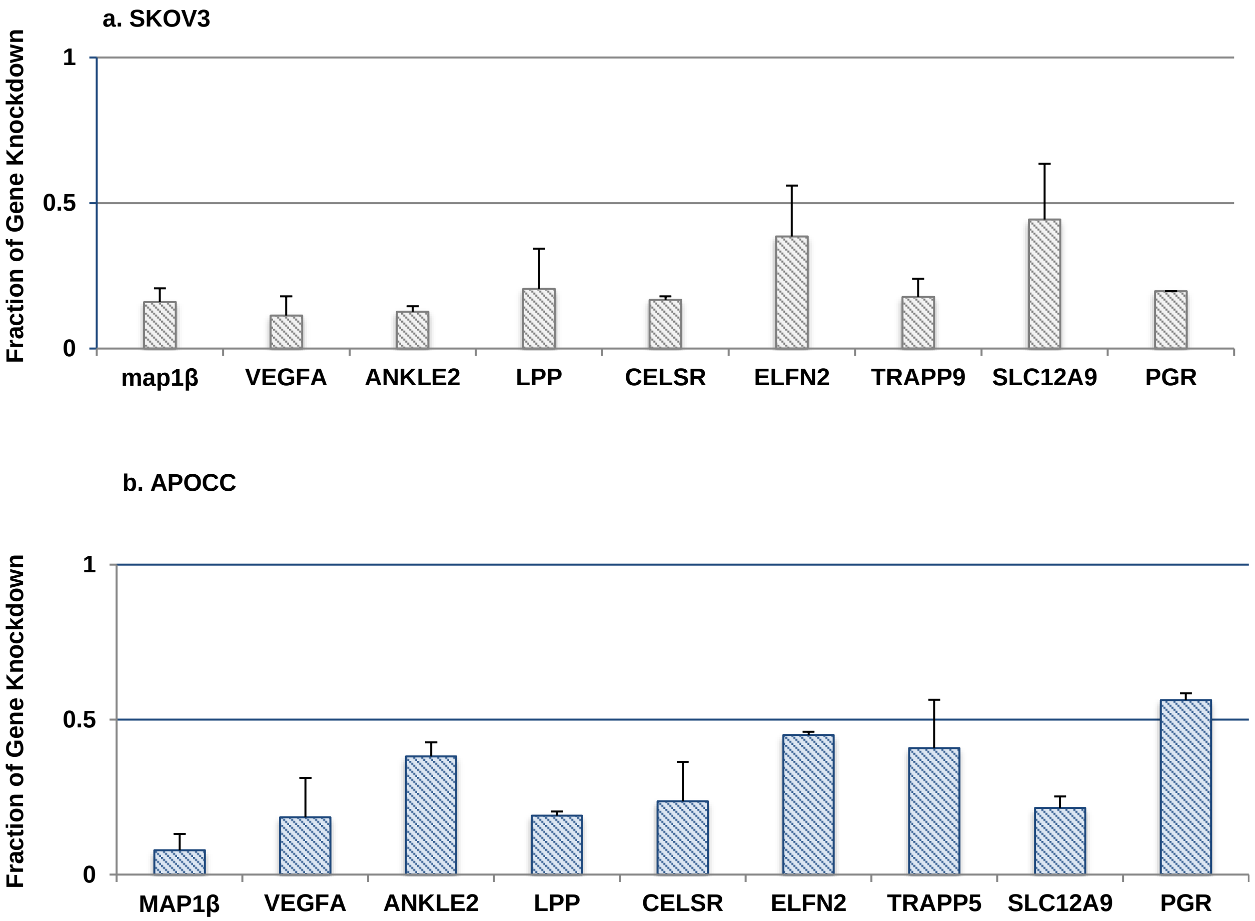

Figure S7: siRNAs Induced mRNA Knockdown with >50% Efficiency as Measured by TaqMan® Gene Expression Assays. (a) SKOV3 and (b) APOCC cells were transfected in 96-well plates with siRNAs (10 nM) with each of 9 different siRNAs. 72 hrs post-transfection, total RNA was isolated and converted to cDNA. Each siRNA was transfected in triplicate. Levels of mRNA remaining after siRNA treatment were assessed using qPCR with TaqMan® Gene Expression Assays. The error bar represents the standard deviation
